# Supplementary figures and images for: CircKIF4A Is a Prognostic Factor and Modulator of Natural Killer/T-Cell Lymphoma Progression
Source: Cancers (Basel). 2022 Oct 9;14(19):4950. doi: 10.3390/cancers14194950 (PMC9562661; doi:10.3390/cancers14194950)

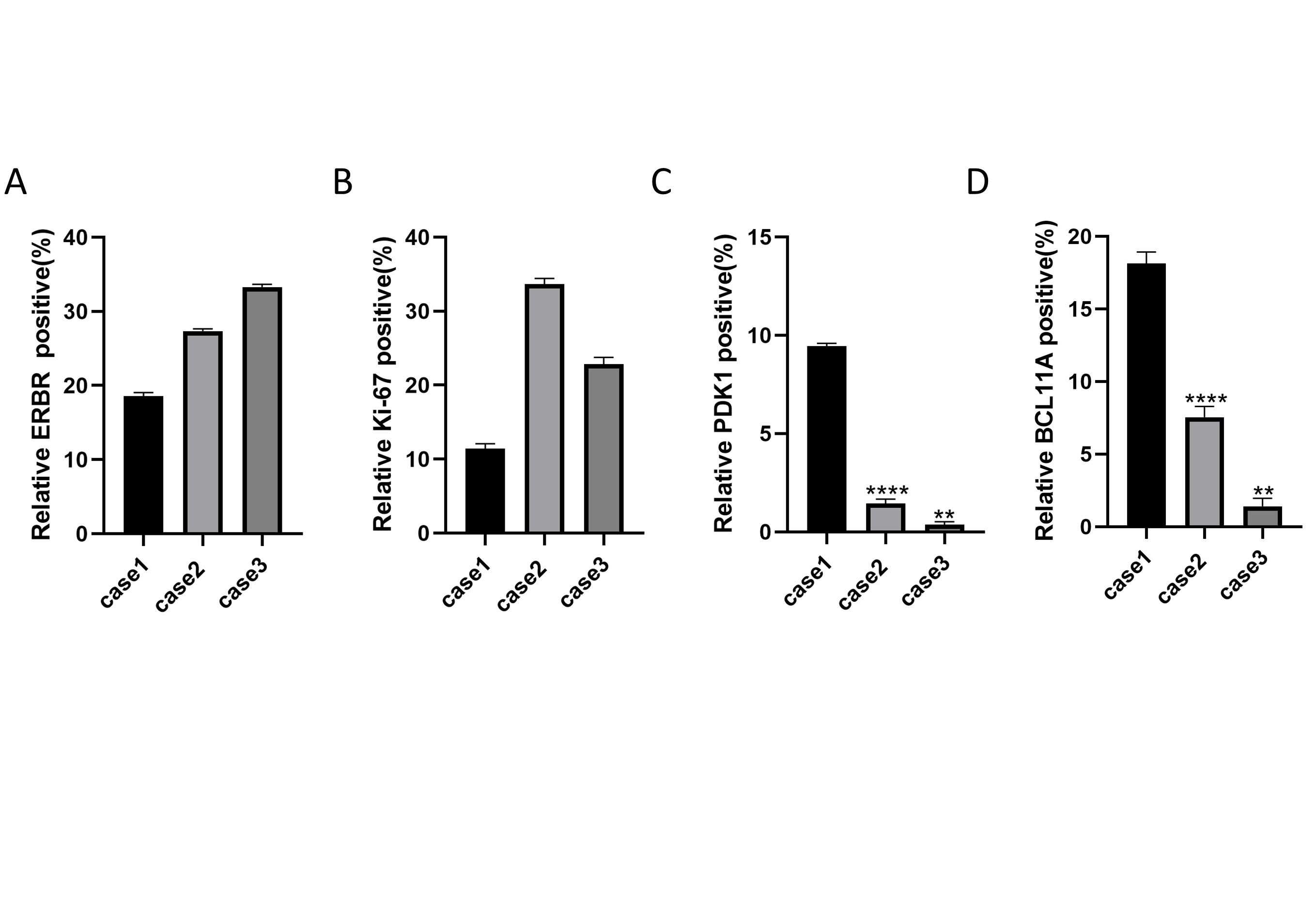

Supplement: Supplementary file 1 [file cancers-14-04950-s001.zip › supplementary Figure S1.tif]
